# Intelligent escalator passenger safety management

**DOI:** 10.1038/s41598-022-09498-x

**Published:** 2022-04-01

**Authors:** Vasily Osipov, Nataly Zhukova, Alexey Subbotin, Petr Glebovskiy, Elena Evnevich

**Affiliations:** 1 Saint-Petersburg Federal Research Centre of the Russian Academy of Sciences, 39, 14 Line, St. Petersburg, 199178 Russia; 2 Saint-Petersburg State Electrotechnical University, Prof. Popov Street 5, St. Petersburg, 197376 Russia

**Keywords:** Computer science, Information technology, Software

## Abstract

This article addresses an approach to intelligent safety control of passengers on escalators. The aim is to improve the accuracy of detecting threatening situations on escalators in the subway to make decisions to prevent threats and eliminate the consequences. The novelty of the approach lies in the complex processing of information from three types of sources (video, audio, sensors) using machine learning methods and recurrent neural networks with controlled elements. The conditions and indicators of safety assurance efficiency are clarified. New methods and algorithms for managing the safety of passengers on escalators are proposed. The architecture of a promising safety software system is developed, and implementation of its components for cloud and fog computing environments is provided. Modeling results confirm the capabilities and advantages of the proposed technological solutions for enhancing the safety of escalator passengers, efficiency of control decision making, and system usability. Due to the proposed solutions, it has become possible to increase the speed of identifying situations 3.5 times and increase the accuracy of their determination by 26%. The efficiency of decision making has increased by almost 30%.

## Introduction

Escalators are essential and indispensable parts of subways and train stations, shopping malls, underground passages and other objects of public infrastructure. Their length can reach 140 m, and the height of the rise can be 70 m. During the operation of escalators, some events can occur that threaten the health and life of passengers^1^. Specifically, it can be a fall into the escalator tunnel, pinching of body parts of small children and animals, equipment malfunction: brakes, electricians, grounding, etc. These events can be caused by sharp stops and breakdowns of escalators and by abnormal behavior^2^ (children, sick and drunk people, etc.): passengers on escalators can stand in an unstable manner, not hold on the handrails, push each other, stumble and fall on the sharp edges of the steps. Escalators can be overloaded, thus creating dangerous situations. All this can result in serious injuries and even death^3^. Dozens of people die every year in such accidents, and injuries are counted in thousands^4,5^.

To reduce the risk of such events, escalators are monitored, as well as the behavior of people using them^6^. In the process of monitoring threatening situations and rules, violations by passengers are recognized in a timely manner. The process of safety control based on the results of monitoring escalators assumes issuing recommendations on the behavior of people, directing flows to parallel lifting and transportation vehicles, and warnings about the stops of escalators. Escalator safety operators, together with technical systems, manage safety to stop the escalator and assist passengers. The operator approach is rather simple but expensive and does not always provide high accuracy and agility of solutions. The use of additional technical means such as video cameras and facial recognition makes it possible to increase the efficiency of passenger safety control^7^.

To reduce the required material resources and to increase the accuracy and speed of solution control, it is desirable to deliver all these functions to promising intelligent systems. The initial information for such intelligent systems can be data from video cameras, microphones and signals transmitted from sensors on the statuses of the escalators. To provide operative safety control, these systems should meet certain requirements. With large passenger flows on escalators, intelligent safety control systems should not only recognize threatening situations in a timely manner but also to do so with a high probability and be able to define appropriate control actions. A review of some existing solutions^8,9^ shows that there is no integrated intelligent approach that proposes a problem of developing more intelligent solutions that are able to take into account the considered specifics of the processes of passenger safety management.

Back in 2016, the problem of developing a strategy for ensuring the safety of passengers on escalators using intelligent means was raised^10^. The issues of improving this safety, its monitoring and management through the intellectualization of these processes were repeatedly discussed at the annual symposia on technologies related to elevators and escalators^11,12^. The main difficulties in ensuring this safety are connected precisely with the imperfection of an integrated approach to processing heterogeneous information both about passengers and the escalators themselves, with the recognition and prediction of complex fuzzy events, with the development of advanced control actions to prevent possible threats. In the interests of this, various artificial intelligence methods are used, based on symbolic, sub-symbolic and combined approaches^13,14^. The most promising approaches include neural network solutions that allow the most complete connection and processing of heterogeneous signals. At the same time, it is necessary that intelligent passenger safety systems on escalators continuously learn and be able to simultaneously successfully recognize and predict threatening situations.

The novelty of the proposed approach lies in the advanced complex processing of information from three types of sources (video, audio, sensors) using machine learning methods and recurrent neural networks with controlled elements. The conditions and indicators of safety assurance efficiency are clarified. New methods and algorithms for managing the safety of passengers on escalators are implemented in a promising safety system in which components are deployed in cloud and fog computing environments.

The article is arranged as follows: the second section is devoted to critical analysis of existing safety methods and to the problem formulation of the development of the intelligent system for managing the safety of passengers on escalators. In the third section, the conditions and indicators of the efficiency of passenger safety assurance are researched and refined. The fourth section addresses the conceptual model for ensuring the safety of passengers. The fifth section describes the passenger safety assurance method. The sixth section presents the algorithms that implement the safety assurance method. The architecture of a complex data processing system based on using fog and cloud computing environments is presented in the seventh section, and proposals for its implementation are presented in the eighth section. Modeling results are discussed in the ninth section. Conclusions are formulated in the tenth section.

## Background

Modern hardware for information processing and control solution implementation is considered in^15–17^. These studies focused on tracking moving objects, posturing people, and predicting emergency braking on escalators. Research^18^ proposes a multimodule integrated safety system for escalators based on computer vision. It considers the problems of escalator status identification, passenger monitoring, detection of hazardous objects, safety assessment and threatening event forecasting. The proposed intelligent system uses convolutional neural networks to process the source information. In^19^, the possibilities of timely warning of potential escalator failures by means of processing signals from vibration sensors on escalators were investigated.

Despite the results obtained, it is not yet possible to implement control functions without escalator operators being involved in the process. Many creative operations of passenger safety control, which are performed easily by operators, can cause significant difficulties under machine implementation. One of the key reasons is the lack of effective methods of intelligent processing of heterogeneous information, which makes it impossible to create full-fledged intelligent systems for passenger safety control on escalators.

To date, a considerable number of complex control systems have been developed. Some of the ideas underlying them can be used to improve passenger safety management systems on escalators. In particular, it is known a safety control system for the cabins of electric train drivers that constitutes a part of the entire control system of the train. Methods of artificial intelligence, pattern and sound recognition are used in these systems. Source information comes both from the sensors and from the alarm and centralization system deployed on the railway. There are separate traffic control services that send signals to railroad points and to semaphores on a particular section of the railway. This is a large integrated system of permits and prohibitions, including a system of safety control and regulation of rail transportation. Examples of such complex traffic and safety control systems in the United States are ACSES, PHW, Wabtec, CBTM, LIRR developed by Bombardier, Parsons Transportation Group (PTG), PHW Inc. These systems comply with FRA, AARS, NORAC, and GCOR standards, which are also used in Spain and Great Britain. In the territory of the European Union (Poland, Germany, France, Italy, etc.) there are analogues of systems used in the USA: ERTMS Regional, TETRA, developed by Banverket and UIC organizations. In Brazil, VLI and ALL companies are the developers of such systems. The systems coordinate train speed with the help of a set of satellites. Satellites are also used to provide safety by space observation for geolocation and rail track assessment. These systems are complexes that include control units where information is collected from locomotive sensors. The units are interconnected into a single network and coordinate with each other's work. There are also auxiliary units for commutation, switching, diagnostics and direct control. Information from diagnostic sensors and coordination services is accumulated on the control panel. The liquid crystal screens display information about the railway (video, rail status, etc.). The locomotive driver is provided with statistics on the condition of the track, so he or she is less anxious and can implement better train control. The system proved to be efficient; during the testing operation of the system, there were no critical cases or emergency incidents, and the system successfully passed the test and deployment stages. The developments of the Krylov State Research Centre (https://krylov-centre.ru/) can serve as an example of a system of this kind in Russia.

The authors propose taking advantage of the existing experience in creating complex systems^20,21^ (in which components interact with each other at different levels of information processing) and creating a system based on previous developments to ensure the safety of passengers on escalators. Unlike existing solutions, it should not be a separate system based on sensors but a comprehensive safety system using intelligent video surveillance and analysis of sound information. In the interests of justifying the requirements of such a system, the conditions for ensuring the safety of passengers on escalators are considered below.

## Escalator safety conditions and performance indicators

### Threats to the safety of passengers and countermeasures to prevent them

All dangerous situations can be divided into three groups. The first group is situations when the escalator is operating in a normal mode. The second group is threatening situations (there are signs that one or more emergency events can occur, for example, indication that the escalator is overloaded or presence of odd noise that means its malfunction). The third group is emergencies (an emergency event occurred, for example, a person fell on the escalator).

A list of possible situations is defined for each group^22^. Each of the above situations may be characterized by the harm done.

General threatening or emergency situations:

1. Falling of a passenger into an escalator tunnel due to negligence or illness;
2. Passenger injury with static electric current;
3. Jamming of the passenger in the receiver of the aircraft handrail;
4. Sticking the feet and other parts of a passenger body into the comb of an escalator;
5. Escalator failure due to exceeding the permissible load per step (more than 300 kg);
6. Failure of the passenger to the office spaces and premises of the escalator in case of failure of the on-board fences;
7. Ignition of escalator parts;
8. Failure of escalator brake and electrical circuit;
9. Drop of lining or plaster on escalator in case of concrete insulation damage.

All the threatening situations listed above can be detected and recognized using escalator automated means (sensors), CCTV (closed-circuit television) cameras and operators of escalator equipment.

Measures to prevent a threat or eliminate its consequences are defined in the form of logical rules (a system of rules defined with relation to a group of escalators).

In the case of a threatening situation, the escalator operator must undertake the following actions depending on the degree of danger:

1. Make a warning via microphone;
2. Stop the escalator and announce the situation;
3. Stop the escalator, close the entrance and ask everyone to enter the station lobby or plat-form;
4. Deenergize the escalator, turn off the main electrical circuit and ask to use protective equipment (gas masks, fire extinguishers, etc.).

### Tools and capabilities for safety status data collection

A wide range of modern sensors can be used to collect data on escalator passenger safety^23^. They can be divided into the following classes:

1. Motion sensors function based on the light flux. They can determine the movement of passengers on the steps, movement of the conveyor belt, and integrity and structure of the steps;
2. Radioactivity sensors detecting radioactive contamination or transportation of radioactive materials;
3. Smoke detectors that can recognize micro particles of burning with high precision: cigarettes smoke, arson of article, clothing and other burning materials. Such violations are committed mainly by adolescents and in the evening;
4. Light sensors: determine light intensity thus enabling the detection of narrow-range low energy blinding lamps which can cause damage to the eyes of passengers and subway employees. They can also detect small-range lasers, video cameras and similar small-range equipment capable of seriously harming people.
5. Fire detectors indicating an open flame.
6. Weight sensors estimate how many objects are placed on one step of the escalator. If the permissible limit is exceeded, the braking system can be switched on. The steps of the escalator are designed so that they can function only within a prescribed load per step.
7. Vibration sensors signal that a group of people create causes a uniform oscillation of the escalator tape. This can cause the escalator to fail and stop at some point. Measures are being undertaken to desynchronize the tape to avoid the peak load. Uniform movement of the handrail that prevents synchronous oscillations and exceeds load is prohibited;
8. Shock sensors detecting equipment damage as a result of either the breakage of escalator parts that strike the moving tape, thus destroying it, or of unauthorized actions such as the use of hammers or other sharp objects that destroy moving parts of the escalator tape;
9. Fall sensors are designed according to a different principle than shock sensors. They detect a smooth oscillation propagation curve, which means the fall on the step of some soft object, a bag or a person;
10. Static voltage sensors show the electric current value, which may occur on the escalator due to, for example, the failure of grounding devices. The peculiarity of large and long escalators consists of their ability to produce high current values in seconds, which is dangerous for the life of passengers;
11. Engine failure sensors can report ignition or crushing of rotation parts or current-carrying parts, which can cause fire or de-energizing of the escalator;
12. Handrail integrity and malfunction sensors can signal about an object under the handrail belt, excess rail speed, handrail fracture or partial destruction of a handrail or tape receiver. Intentional cuttings of the handrail are not uncommon. A long handrail with high tension can cause serious injuries to passengers if torn;
13. Out-of-sync sensors show the dynamics of oscillation changes between all steps of the escalator, its handrails and other parts of the equipment. Out-of-sync is necessary to reduce the peak load and to avoid escalator destruction;
14. Speed sensors monitor the limit of speed of moving part rotation. Exceeding the speed admissible limits may cause serious consequences, even for human victims when passengers die after falling on the platform or escalator steps in the case of a brake system failure;
15. Pulse sensors make it possible to detect the need for repair of the escalator due to wear of steps and damage to connections.

Sensors are not the only data-producing devices; an important source of data about the escalator is technological microphones. There are microphones that are de-signed in such a way that the audibility of sound (amplitude of sound pressure) is the same regardless of the distance: 10 m, 50 m, 100 m. Filtering is carried out according to selected frequencies: voice, mechanisms, etc., which helps to eliminate noise and to listen only to the target frequencies. Such audio streams can be stored in a computer system for subsequent analysis.

All-weather high-resolution video cameras with additional detailing are also used in the subway. Video streams from them can be stored for a short time and for a long time in a computing system using storage servers. Based on their analysis, the current statuses of objects under monitoring can be recognized.

### Safety assurance indicators and possible ways to enhance the values

Upper-level indicators may be assessed in terms of cases of prevented passenger health injuries (psychological, physical and others)^24,25^.

These indicators depend on a number of passengers, the time period under consideration and on the safety system efficiency; the latter can be assessed by the speed and accuracy of identifying and forecasting threatening situations and by the timely formulation and implementation of appropriate control measures for changing the transportation process and passenger behavior. The speed and accuracy of threat detection and forecasting and the efficiency and adequacy of safety control solutions depend to a large extent on the methods of information collection and processing and on the software and technological tools being used. Various methods provide different capabilities for processing video, audio streams and other signals from sensors. It is desirable to find and use appropriate methods to achieve the set goals while meeting a number of objective conditions. It is clear that passenger safety assurance is subject to the characteristics and quality of data on the status of the process under control being obtained from sensors of different kinds. It is desirable that the sensors provide complete, timely and accurate information.

In particular, the accuracy and speed of receiving information about passenger safety can be enhanced through the use of cloud and fog computing. Due to the high performance of cloud systems, it becomes possible to use more complex algorithms that provide high accuracy of object recognition in video images but have stronger requirements for computing resources.

The high performance of modern cloud and fog systems allows the processing of video streams together with audio streams using artificial neural networks. Such joint data processing results in significant enhancement with regard to the accuracy of threat detection. In fog and cloud systems where sensor information is processed simultaneously with audio and video information, the accuracy of determining threatening situations can be increased by tens of percent compared to conventional solutions.

The speed of detecting threatening situations can also be increased by developing and introducing a special mobile application for smart phones of safety control operators. Such an application gives the smart phone owner the possibility to have information about events that are registered by various systems and by other subway employees. In the case of a threatening situation, subway employees will be able to enter the mobile application using one key press and evaluate the situation on the platform, lobby or escalator. Taking advantage of the developed mobile application that receives status signals from a complex system, it is also possible to increase the efficiency of decision making. A new mobile application will increase the responsiveness of staff, ensuring the prompt exchange of information between the operators of the escalator and CCTV. In addition, a new approach allows the saving of human resources. A person cannot be in a state of constant tension for a long time, which leads to nervous system exhaustion. One of the advantages of the proposed approach is the reduction of the active involvement of employees in routine activities. Auctioning of the staff is required only at the critical periods when the system identifies threatening situations and requires decision making.

## Conceptual safety model

**Figure 1:**
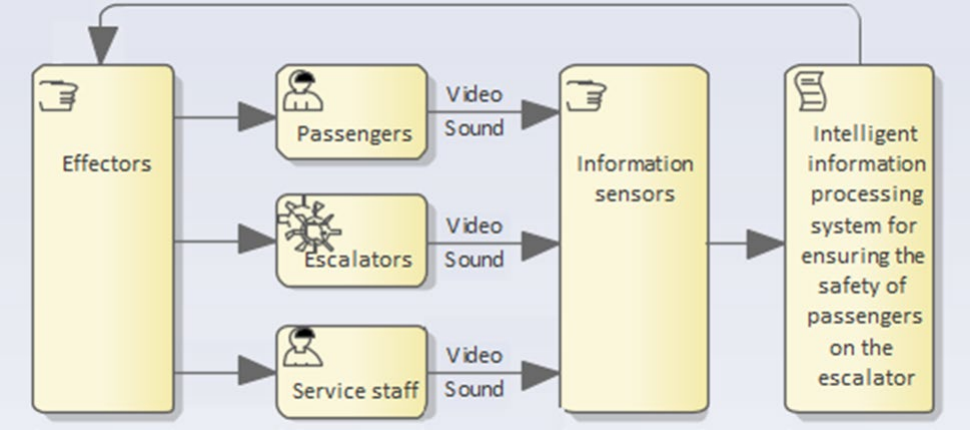
Conceptual safety model.

To develop a comprehensive method and advanced passenger safety system, some considerations are first given to a conceptual model that ensures the required level of safety (Fig. 1). The main idea of the proposed conceptual model is to organize joint processing of data received from various sources to recognize threatening situations in time.

Passenger safety on the escalator is ensured by processing collected data in an intelligent information processing system in several contours. The contour of information processing from sensors makes it possible to provide escalator operators and safety responsible staff with a "basic picture" of emerging situations. Data to be processed in the first turn are those obtained from radioactivity, smoke and static voltage detection sensors. Processing and analysis of video information helps to identify inadequate behavior of a passenger or a group of passengers. Audio information processing completes the formation of a "safety picture", which gives the operator a comprehensive representation of the events taking place on the escalator, for example, equipment failure, fire, terrorist threat or hooligan action.

Data processing is carried out in a closed loop in one or more contours wherein both data of the same type and of different types can be processed; for example, data from sensors and video information can be combined, or all three types of data—data from sensors, video and audio information—can be treated simultaneously. Data processing results obtained in different contours comply with each other. All implemented processing actions are interconnected, thus forming a single process aimed at obtaining information ensuring passenger safety.

In the case of a malfunction of one of the data collection systems, for example, video cameras, the safety control operator may receive operational data from sensors. In the case of sensor failure, data transmitted from process microphones can be used.

For data processing, artificial neural networks and machine learning methods can be used. For example, video stream processing methods of artificial intelligence being applied to the sequence of frames are capable of detecting objects that can affect the safety of passengers, while the identification problem is solved taking into account the context that is determined by the data from other sources. Solving the identification problem in such a statement, as a rule, requires the use of several neural network methods, a significant part of which includes complex calculations.

Application of the proposed conceptual safety model makes it possible to increase the validity of the generated results as well as the reliability of passenger safety systems.

New passenger safety systems based on joint data processing can offer solutions for the following new classes of problems:

- Prevention of terrorist acts and hooligan acts;
- Provision of timely assistance to the passenger in case of a sharp deterioration of health;
- Ensuring safety of the passenger in case of malfunction of escalator equipment or its parts (electricity, mechanics, etc.);
- Timely assistance to low-mobility passengers (disabled persons);
- Registration of a chronology of events in case of an emergency.

To solve the above problems, the system can propose the following solutions depending on the gravity of the situation and the currently available means to fix it:

- Call an operator to assist a disabled person in case of his inability to move independently and absence of an accompanying specialist;
- Stop the escalator in case of items stuck, in particular shoes, clothes or body parts in escalator equipment mechanisms (comb, side parts of balustrade, handrails, etc.);
- Call an ambulance if the passenger falls from the escalator and is injured;
- Call the police and special services in case of a terrorist threat;
- Call the firefighter, stop the escalator, turn on the fire prevention system, de-energize the escalator in case of smoke, fire or open burning, previously persuading in the high degree of danger of the situation that has arisen, i.e., making sure that the smoke is not the result of cigarette smoking or the actions of a minor bully who has set fire to an article or other objects;
- Deenergize the escalator and call emergency services in case of electrostatic failure;
- Switch on the backup brake system in case of failure of the main brakes or damage of the escalator engine or side handrails.

## Passenger safety assurance method

The process of ensuring passenger safety on the escalator is based on the complex processing of data obtained from surveillance cameras, from technological microphones and from diverse sensors^26^. Collected information is processed by the system using artificial intelligence methods. These methods include recognition of signals, objects, situations, forecasting of potential consequences, intelligent development and implementation of control solutions. If the situation is insecure, a so-called signal status is generated. The status signal is an information message that contains data on the current situation and its type (secure/threatening/emergency). It can be processed both inside the intelligent technical passenger safety control system and sent to the smartphone of the safety control operator.

The safety operator, having received a signal status informing about the occurrence of a threatening or emergency situation, can act in three different ways.

The first way is to take control to help passengers take responsibility. In this case, the operator of the technical system evaluates the situation and makes decisions on measures to prevent passenger safety threats. He or she may also share responsibility with senior operators and various subway services. The second method involves monitoring the operation of an intelligent technical system, making changes in its solutions or approving them. The third way is to pass all the control functions to the intelligent system.

**Figure 2:**
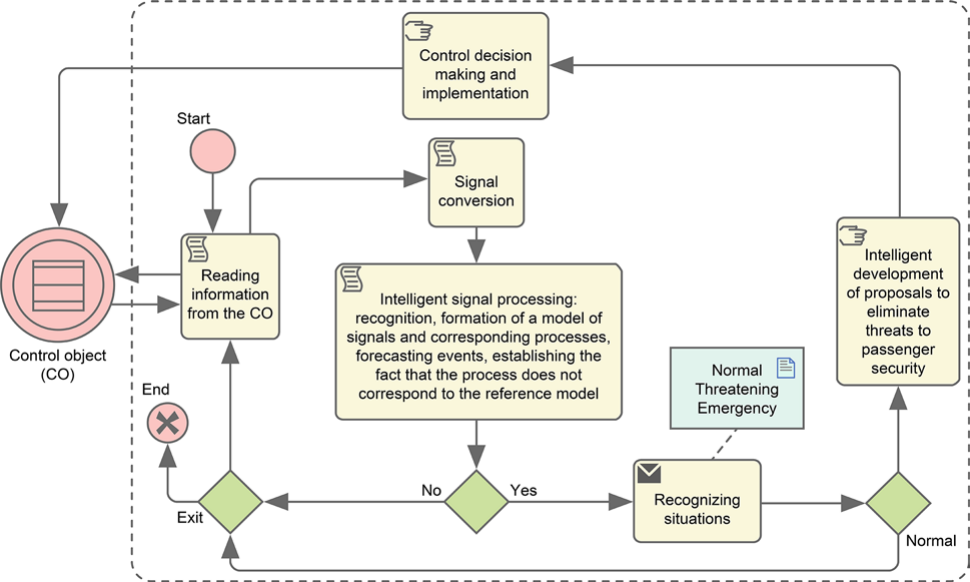
The process of intelligent passenger safety control on escalators.

The process of intelligent passenger safety control on escalators is shown in Fig. 2.

Passenger safety systems, realizing the process shown in Fig. 2, also provide records of events, situations that have arisen and the details of their development (date, time, type of event, level of danger, place, etc.), circumstances under which the situations have arisen, services involved in situation elimination and potential negative consequences.

**Figure 3:**
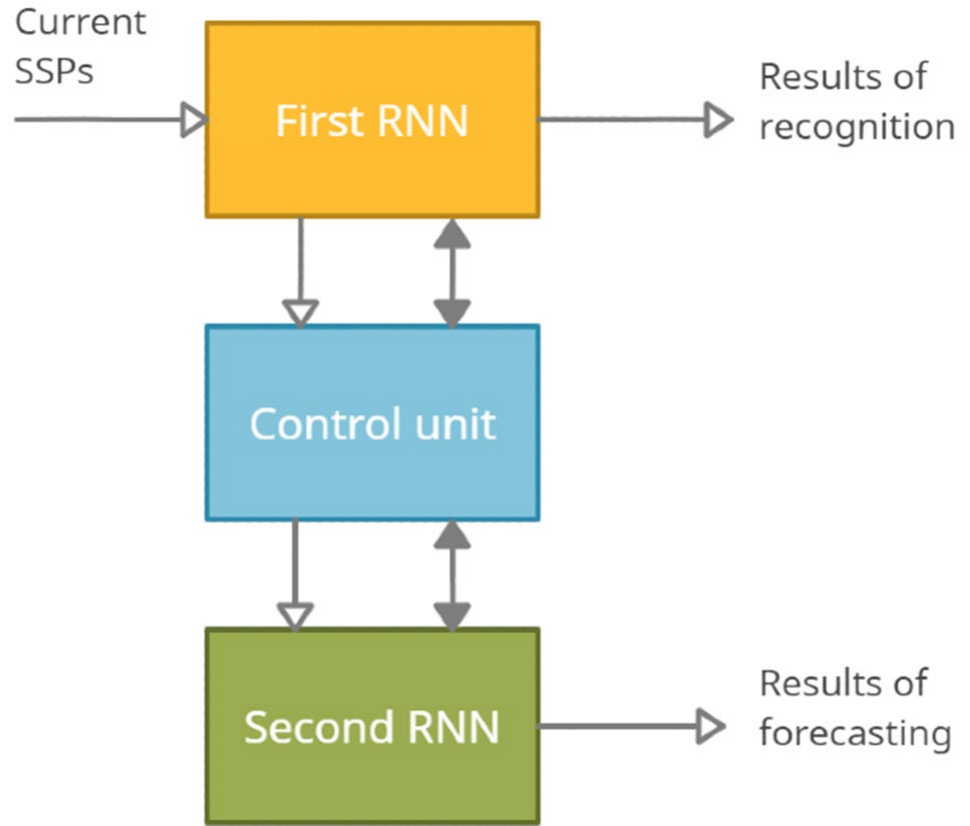
A neural network system with continuous learning for recognizing and predicting events: RNN—recurrent neural network; SSPs—sequences of the sets of single pulses.

For intelligent signal processing targeted at solving problems of recognition and forecasting of events, it is proposed to use a neural network system^27–29^, the generalized structure of which is shown in Fig. 3.

This system consists of two identical recurrent neural networks and a control unit (Fig. 3). For the perception of signals from sensors by this system, they must be converted into sequences of the sets of single pulses (SSPs). In the general case, each original signal can be decomposed into spatial-frequency components. In this case, each component can be transformed into a sequence of single pulses with the repetition rate and phase as functions of the amplitude and phase of the component. As a result, when considering a process in discrete time, we have a sequence of sets of single pulses.

The first RNN is responsible for the continuous formation and storage of the model of input signals and the results of their recognition. In this RNN, the formation of a model of input signals is implemented by binding them on network elements, taking into account previously stored information. The continuously updated neural network building model provides the most complete identification and memorization of patterns inherent in input signals. Recognition of signals and their characteristic events using this model comes down to binding them with known memorized facts and extracting them from associative memory. Prediction based on this model is feasible with an accelerated call from associative memory of future events by processed signals. To implement such a prediction without interrupting the learning process of the first RNN, the second RNN is used.

**Figure 4:**
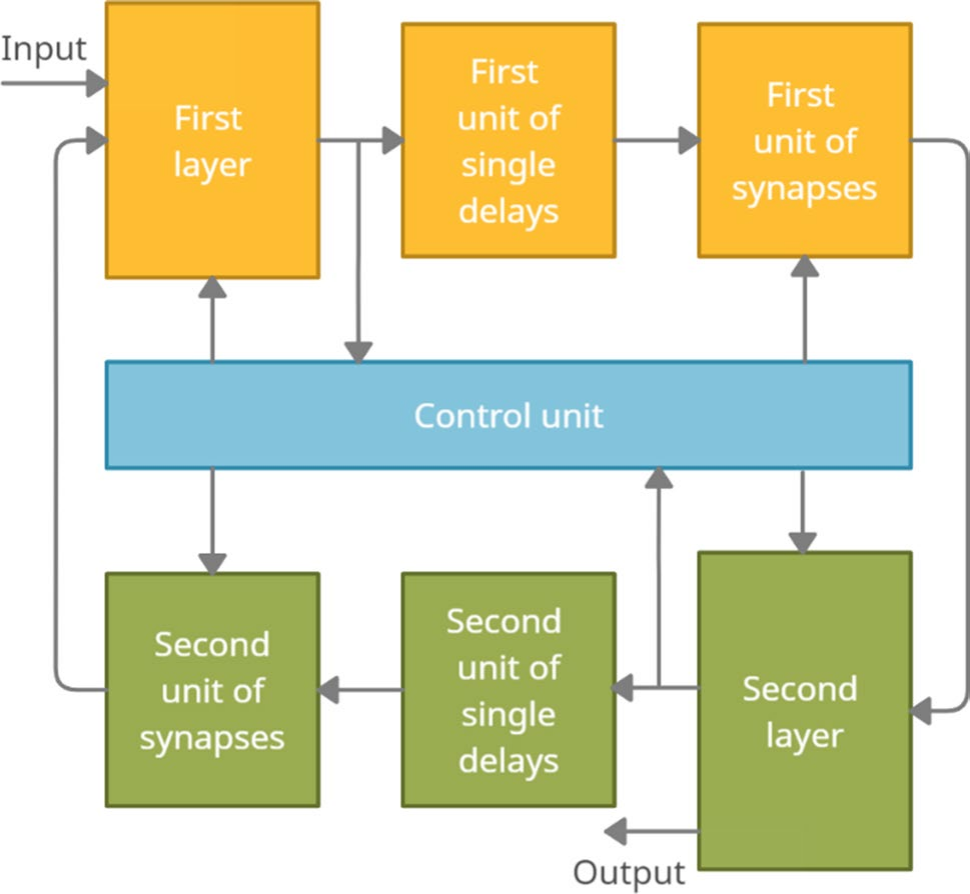
Flow diagram of a recurrent neural network.

The second RNN is used only for predicting events. In the interest of prediction, the control unit periodically reads the status information of the first RNN into the second RNN. After reading this information in an accelerated time, the operation of the second RNN and the formation of the forecast begin. To improve the prediction accuracy on short and noisy signal samples, the RNN provides the possibility of their restoration by controlling the directionality of the associative recall of signals from memory. The diagram of the RNNs^30,31^ used is shown in Fig. 4.

**Figure 5:**
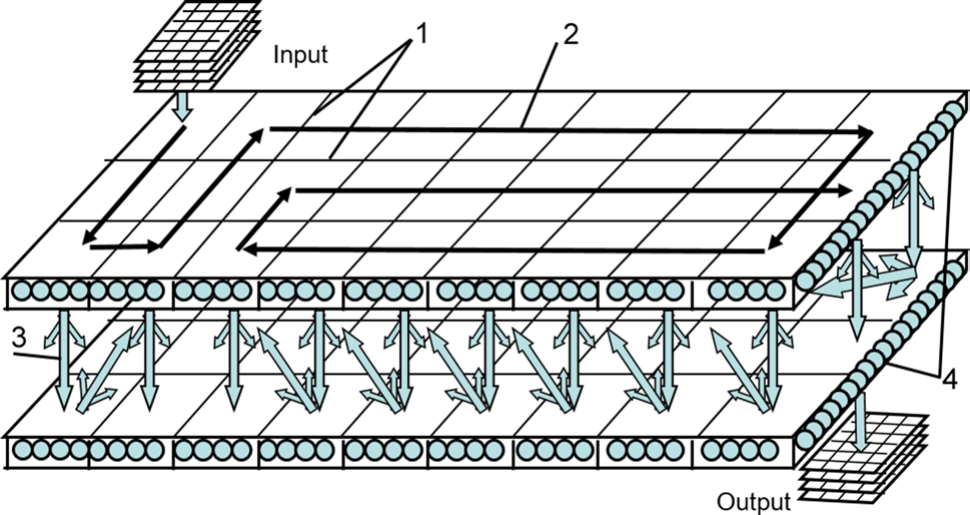
An example of a looped logical structure of a recurrent neural network: 1—lines of dividing network layers into logical fields; 2, 3—directions of movement of the sets of single pulses along and between the layers; 4—neurons.

The features of this recurrent neural network are as follows (Fig. 4). Due to the spatial shifts of the sets of single pulses transmitted from layer to layer, this network can be endowed with various logical structures (linear, spiral, loop, and others). This provides an extended spatiotemporal linking of signals in associative memory. Due to the priority of strong ties, a one-to-one correspondence between the entry and exit elements is realized. An example of the loop structure of such an RNN is shown in Fig. 5. Other structures can be found in^27–30^, which are the author's previous works.

Undoubtedly, the size of logical fields in this neural network is determined by the size of the input SSPs, which can be composited and carry simultaneous information about many initial signals from sensors (Fig. 5). Note that sequences of sets of single pulses at the RNN outputs can be converted into corresponding original signals.

The use of such solutions for intelligent processing of signals from sensors in the interests of ensuring the safety of passengers on escalators makes it possible to expand the possibilities of both recognition and prediction of threatening events.

## Solution algorithms

**Figure 6:**
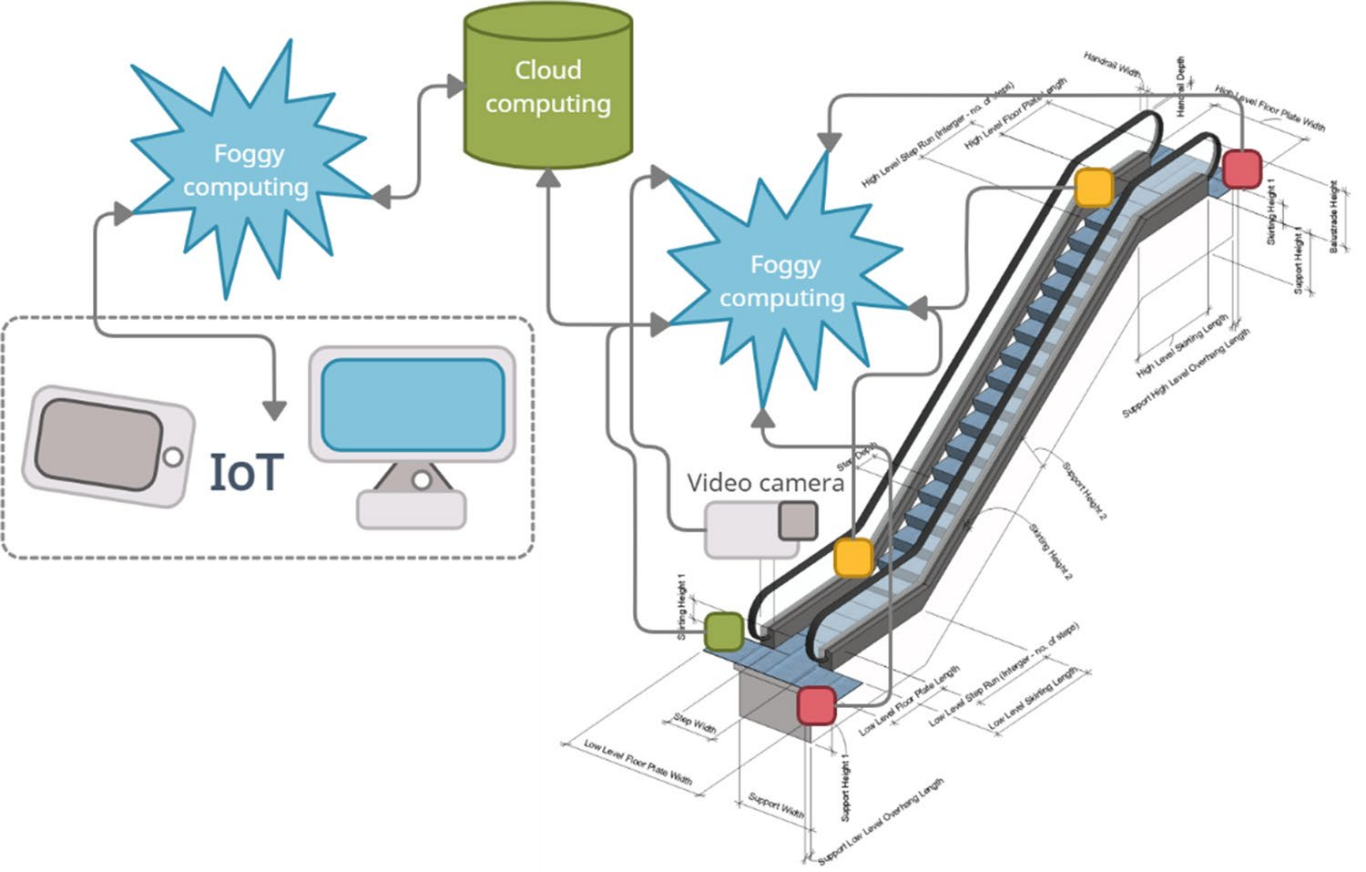
Logic structure of a system functioning in a fog computing environment.

To ensure the safety of passengers, it is proposed to develop an intelligent system that provides joint processing of data from various sources^32–34^. The general functioning scheme of the system is shown in Fig. 6. The system assumes wide usage of the Internet of Things (IoT), fog and cloud computing technologies^35–37^.

Joint data processing in the system is implemented through the use of fog and cloud computing technologies. Cloud technologies enhance information processing performance by several times, and the use of fog environments reduces delays to a significant extent. As a result, the use of fog and cloud technologies ensures data processing by machine learning methods and neural networks, providing necessary performance for the timely detection of threatening and emergency situations.

Data processing in the safety system involves usage of specially designed algorithms (deep learning, convolutional and recurrent neural networks, etc.) for the processing of certain types of data, in particular video and audio information, sensor data. These algorithms are combined in the frame of the general method of joint processing of data received from three sources.

Data collected from video cameras, microphones, analogous and discrete sensors are processed in the cloud. For their processing, ready-made machine learning models and neural networks are used, and new models are being developed. The selection and creation of machine learning and neural network models is controlled from a fog environment by generating scripts based on the characteristics of the obtained data (image characteristics, sound sampling rate, etc.) and transmitting scripts together with the collected data to the cloud.

The video processing algorithm involves the following steps:

1. Transmission of a group of frames from a fog environment to the cloud environment;
2. Dynamic Python script editing to build machine learning and neural network models and transfer them to the cloud; parameters of collected data (dimensions of images, names and paths, format) are transferred together with the scripts;
3. Selection of a profile for machine learning and neural network learning in the cloud, containing prepared in advance image bases for building models according to the list of keywords;
4. Dynamic editing of the image training base and creation of additional models taking into account illumination, weather conditions (frost, haze, high light radiation, rain and snow);
5. Temporary additional training on the cloud server in case of a strong change in the angle of the video camera;
6. Search for the objects in images;
7. Identification of objects using images shot from different angles and by different surveillance cameras;
8. Dynamic selection of models that were built in the cloud in advance using a large set of images for their construction and taking into account the experts’ opinion. Selection is made according to a keyword or a list of keywords.
9. Calculation of the refined probability of finding an object and transfer of information to a fog computing environment.

The logic of audio information processing is similar, but instead of images, the fragments of audio recordings lasting 2–3 s are transferred to the cloud.

The algorithm of processing data from sensors is similar to the processing of audio information, since at the physical level, information obtained from sensors is analogous and is represented in the form of a sinusoid, similar to audio information. Exception is made only for discrete sensors.

Configuration of storage for individual images under framing a video stream and storage for log files and debug information is performed once during program installation.

Installation and configuring of drivers for special technological microphones, connecting audio streams in a program installed in a fog environment is also carried out once when installing the program.

The method of complex data processing based on algorithms for processing data from single sources involves the following steps:

1. Estimation of the probability of occurrence of a certain event by means of processing information from sensors;
2. Refinement of the information about the event on the basis of the data from three information sources: video, audio, sensors; output of a message about threat level, location, frame and sound to the operator's smartphone.
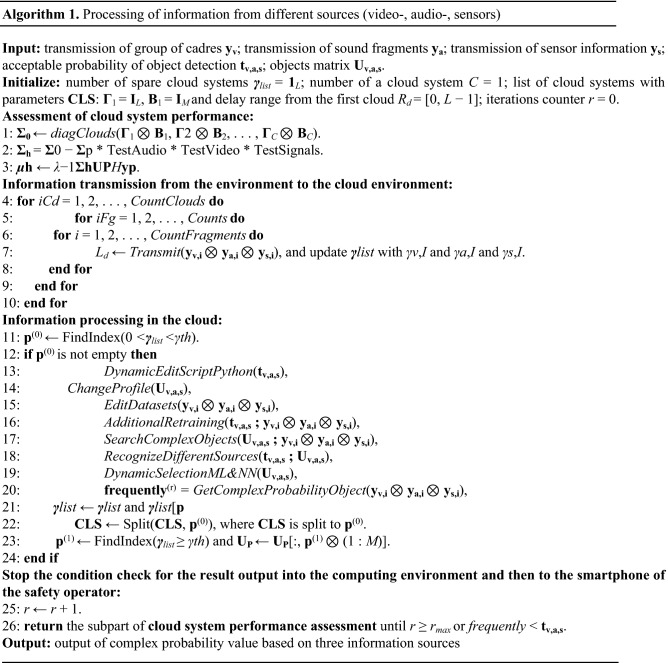

## System architecture

The proposed data processing system has a three-level architecture:

1. End user application is installed on smartphones under iOS or Android;
2. The program for data processing control is deployed in a fog computing environment;
3. Data processing program is deployed in the cloud.

Processing of all types of data takes place in the cloud using the high-performance system Microsoft Azure; obtaining data characteristics necessary for control and carrying necessary data preprocessing is performed in a fog environment, and the result is delivered to the safety operator’s smartphone or tablet. Cloud technologies involve the use of the control console (through a website or command line), which simplifies to a large extent the deployment of applications. Options for selection and configuration of different versions of operation systems (Windows, Linux, Mac, FreeBSD, CentOS, etc.), necessary software (various web servers, machine learning libraries, network administration programs, remote control, etc.) are provided via control console. At the fog level, virtual machines are used, which, unlike the traditional VMware Workstation (https://www.vmware. com/), Virtual PC from Microsoft, can be adapted to certain technical means (taking into account storage parameters and systems, chipsets, central and graphics processors). Adaptation to hardware parameters enhances the efficiency of virtual machine resources by the users of applications.

Ready-made servers can be adapted to solve certain problems in the fog environment. For example, a Cisco UCS C480 ML M5 server that differs from a user home computer by price (20 or even 30 times more expensive) and by pre-installed software, necessary drivers, components (discrete and analog input boards for video, audio, sensors), has a processor with a powerful NVIDIA Tesla V100 RAM graphics core, 96 GB of RAM, a control console that optimizes the resource consumption of a fog computing environment, is offered for use for solving tasks using machine learning and neural networks. Servers that are adapted for other tasks (production process control, banking operations, etc.) are al-so provided.

## System implementation

The application for Android was developed in Android Studio 4.2.1 (https://developer.android.com/studio), provided by Google. The development environment is widely used by programmers because it is free. The new Dart programming language, which is cross-platform, allows the use of developed applications for iOS. In addition, the Dart language is supported by Visual Studio from Microsoft under Windows 10, as well as IntelliJ IDEA under the Linux family of operating systems.

The control program was developed in Java in the NetBeans 12.4 environment (https://netbeans.apache. org). Java supports many network protocols and provides cross-platform features and easy portability of applications. The developed control pro-gram interacts with video cameras and other means used for safety data collection. The work is organized according to the principle of signals: if a signal comes from one device, then another device or program in the system begins to work. For example, a video camera driver transmits a signal status to a fog environment in which the video camera is working. The program opens a stream of video information and begins reading, decoding and story boarding. Audio and video flow processing follows the principle that if one cloud server does not provide high data processing performance, then other servers with higher performance are to be involved in the processing.

Data from the fog environment are transferred to the cloud under the HTTP protocol since this protocol is supported in almost all networks. Information exchange takes place in XML format, and data are loaded in JSON format using web-based technologies PHP, JavaScript, JQuery. The choice of these technologies set was due to the ease of use, relatively low cost of development, the openness of the community of software developers and great popularity, presence of a large number of examples to solve the problems of data exchange and good documentation. The program for the cloud was developed in Qt Designer 5.15 (https://www.qt.io). Version 5.15 is selected because version 6.1 is not stable enough at the moment. Development was carried out in C +  + using TensorFlow library 2.5.0 (https://www.tensorflow.org), which is installed separately or through the Qt pack-age manager.

Data and processing results are stored in the Oracle DBMS, which is located in 64 TB of cloud storage and can be extended. Interaction with the database takes place using the Qt QOCI driver, and it is possible to make connections to other databases through the drivers QPSQL, QODBC, QMYSQL, QDB2, QIBASE, QTDS, and save a temporary data array (2–3 weeks) in the cloud. The application of Oracle DBMS in Azure Storage accounts for the fact that its performance is higher under frequent INSERT requests. In addition, Oracle DBMS has many optimizations for various technical means, as well as for various complex and frequently executed requests.

## Case study

The system developed by the authors was used to monitor the safety of passengers on the escalators and to provide assistance if necessary.

The performance of the system was assessed by the following indicators:

1. Information processing performance;
2. Accuracy of situation identification;
3. Operativeness of decision-making and assistance;
4. Satisfaction with the system (usability, ergonomics, etc.).

**Figure 7:**
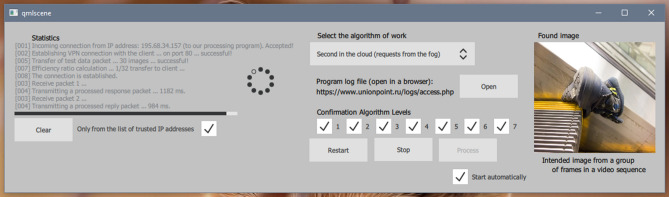
User interface of the program deployed in the cloud computing environment.

The developed system includes three programs. The first program is located in the cloud (Fig. 7). The program was installed on a Microsoft Azure cloud server; network access was configured through the administrator panel. Using the administrator panel (access through the Remote Desktop Connection Manager (RDCMan)), the remote desktop was also configured, and all the necessary cores and processor types, RAM, operating system characteristics and preinstalled software were selected.

The user interface of the program that runs in the cloud and deals directly with image processing is shown in Fig. 7. On the left side of the graphical form of the program (Fig. 7), the statistics of connections to the cloud are shown: IP address, attempt to establish a VPN connection (successful or unsuccessful), the status of data delivery and data transfer process, etc. There is a Clear button for clearing statistics and a checkbox; when it is selected, connections are made only to addresses included in the list of valid addresses, which are configured in the config.ini and settings.ini configuration files in the directory of the application.

In the center of the graphical form, a panel for algorithm selection is placed. For example, as shown in Fig. 7, an algorithm that allows receiving requests from a program installed in the fog environment can be selected. The program also provides logging with the ability to view logs through the web interface. Clicking the Open button opens the browser page, which displays all the events in the system registered in logs. There are data processing process control buttons: Restart, Stop, Process, and a checkbox to automatically start the process; the statuses of the processes are saved in the config.ini file in the program directory. On the right side of the form, the last found object and additional information about the image are displayed.

**Figure 8:**
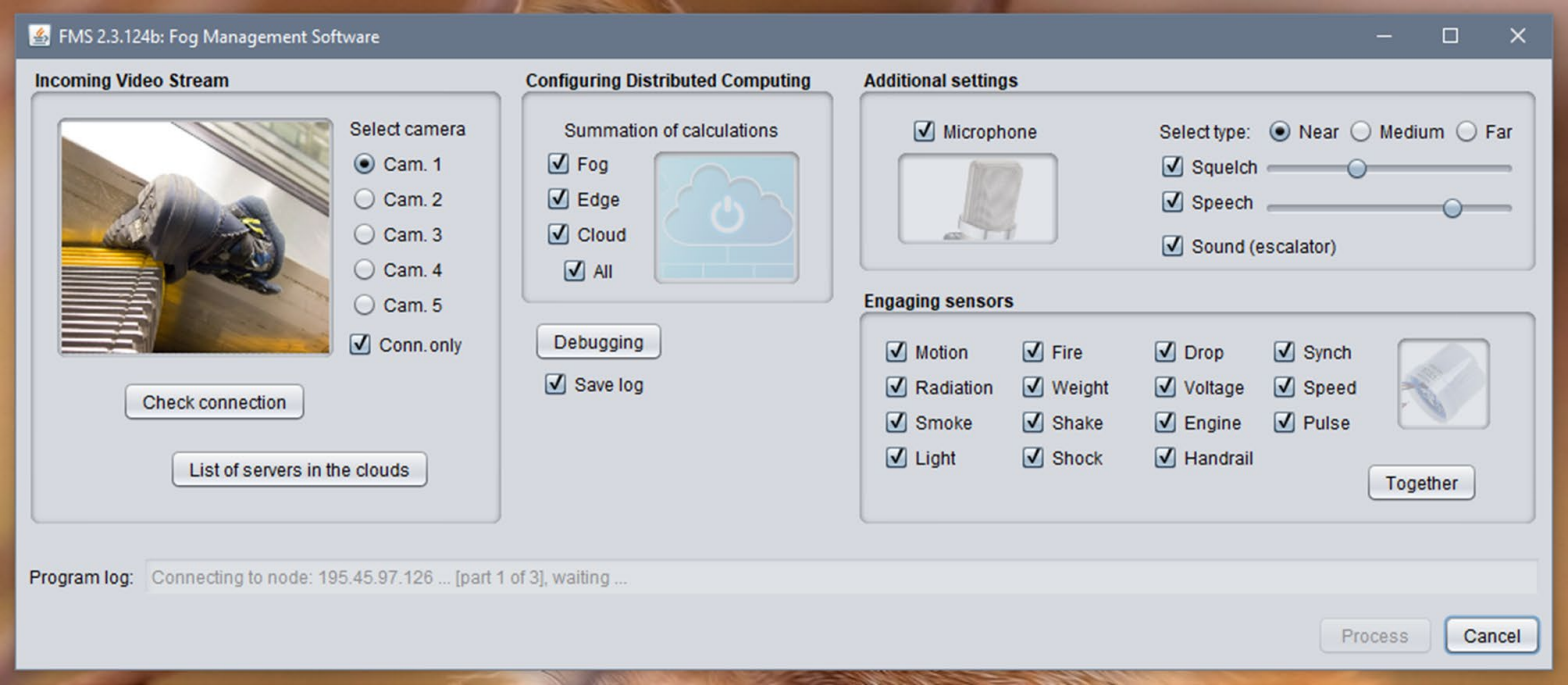
User interface of the program deployed in the fog computing environment.

The second program coordinates the work of the system (Fig. 8), and it plays the role of the information flow manager. The program was developed in Java and deployed in the fog computing environment. While processing video data, the program decodes the video stream from one camera to 12 frames per second (using settings, it is possible to increase or decrease the number of frames), and a package with images is sent to the cloud every 4 s. Frames have the format Full HD with a resolution of 1920 × 1080. In the fog environment, the storyboard of a video stream can process information from 5 cameras, not more.

The user interface of the program that operates in the fog environment and controls the transmission of information flow from three data sources to the cloud is shown in Fig. 8. On the right side of the main graphical form of the program, the video image obtained from one of the surveillance cameras is presented. The camera is selected from the list located on the right of the image (according to Fig. 8, only five CCTV cameras are active: Cam. 1–5). The checkbox ‘Connected only’ allows display only active cameras, and cameras that are not connected are hidden. It is possible to connect additional cameras through the config.ini and settings.ini configuration files in the program directory. At the bottom of the form, there is a button for checking the connection of video cameras "Check Connection" and a button "List of servers in the clouds," which allows download a list of all cloud servers with logins, passwords, etc., access settings. At the bottom of the form, information is displayed about the status of the data transfer process, connection of the fog environment with the cloud, errors, etc., diagnostic messages.

In the center and to the right side of the form, there are panels for the following data processing settings:

1. Computing environments: Edge, Cloud and All;
2. Parameters of microphones: range (near, medium, far), noise reduction, sensitivity, frequency filtering;
3. Set of sensors (Motion, Radiation, Smoke, Light, Fire, Weight, Shake, Shock, Drop, Voltage, Engine, Handrail, Synch, Speed, Pulse), it is possible to select all or only separate sensors (Together button).

It is possible to maintain a debug log that can be turned on and off using the Debugging button; to record logs, the "Save log" switch is provided. At the bottom of the form, there are process and cancel buttons to start and stop the processes of sending data to cloud programs.

**Figure 9:**
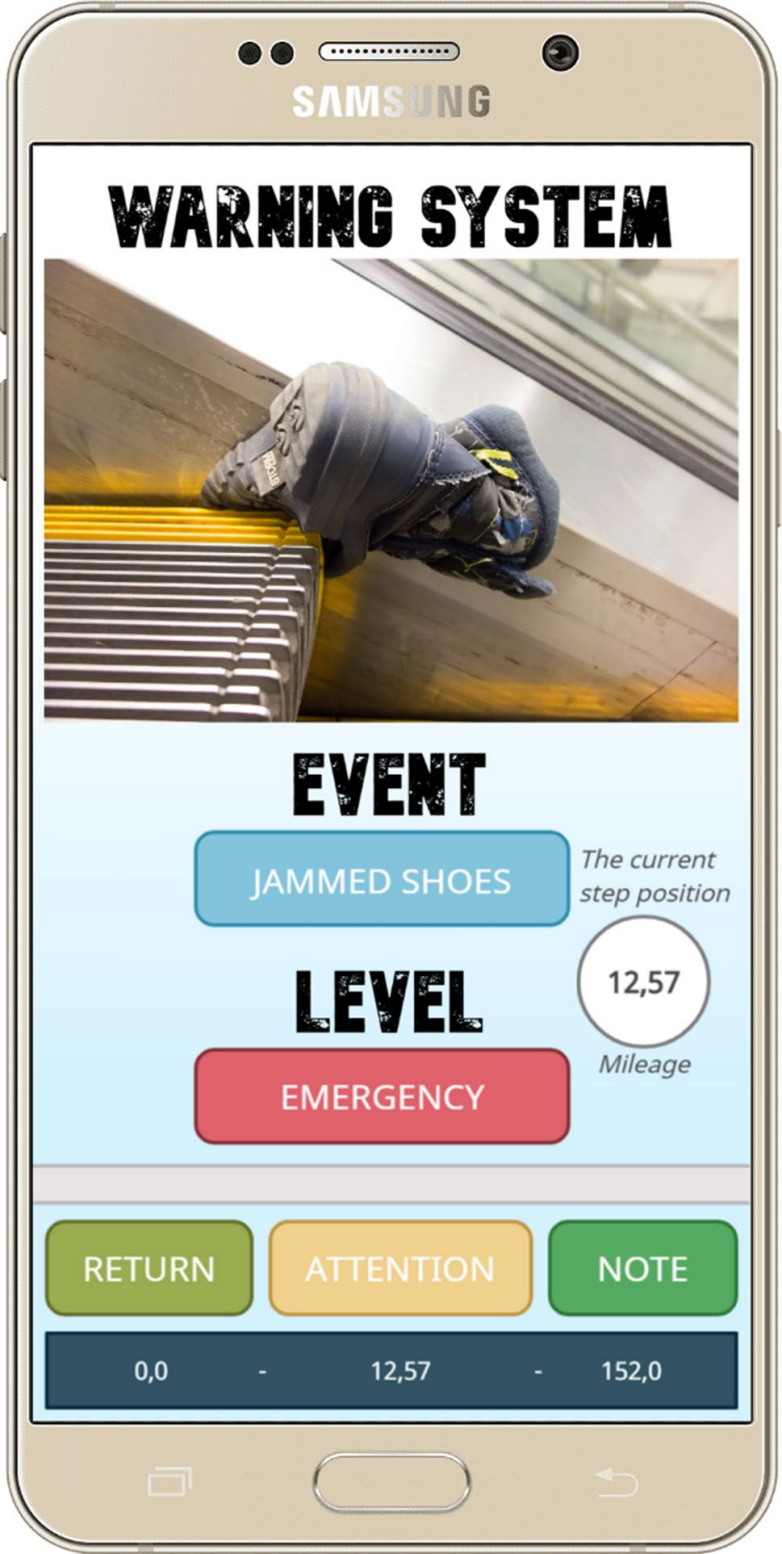
User interface of the application for the operator’s smartphone.

The third program displays information on the situation for the operator in a user-friendly and up-to-date form (Fig. 9).

At the center of the smartphone screen (Fig. 9), a diagnostic message on the danger level is displayed. The control operator can mark the message as ATTENTION and add a comment (NOTE). The RETURN button returns to the main menu. To assess the situation on the escalator, the operator is provided with information about the position of the escalator (at the bottom of the screen) and an image of the escalator (at the top of the screen).

The developed system was used for experimental research of the proposed method for monitoring the safety of passengers on escalators. To replicate the results of the research, it is necessary to connect all of the listed sensors, video cameras and microphones to the expansion cards of the Cisco UCS C480 ML M5 server in the foggy environment, register on Microsoft Azure, select an instance of D2 v3 and start the virtual machine, connect via Microsoft's RDCMan 2.7 remote desktop and install program for the cloud. The same should be done in the foggy environment. On a Samsung Galaxy J3 phone (or any other phone with similar specifications to Android OS, HD sAMOLED 5.0" screen, 4-core processor, and 8 GB memory) in developer mode, it is necessary to copy and install the Android application. Copy the processing time of the information of each package from the text logs of the programs and paste into Excel, apply filtering and perform aggregation (autosum, average, etc.), fill in the results in tables.

The results of experimental studies showed that video processing in a fog environment using cloud technologies increased the speed of detecting objects in images by an average of 3.6 times (Table 1). The columns in Table 1 indicate the time (milliseconds) of image processing in fog, edge, cloud and all, and the last column shows how many times more relative to all and fog.

**Table 1:** Assessment of video processing (object detection) performance.

Object type and number		Fog	Edge	Cloud	All	Speed increase
Footwear clasping	951	145	3178	47	43	3.37
Objects stuck in the comb	739	164	4109	43	41	4
Pinching of objects and body parts with handrail	372	106	2416	32	31	3.42
Getting into handrail receiver	483	152	4059	38	37	4.11
Smoking	571	133	3724	41	40	3.33
Alcohol consuming	794	146	3056	39	37	3.95
Fall at stages	587	165	4124	46	43	3.84
Ignition	209	122	2937	37	35	3.49
Smoke	174	138	2970	42	41	3.37
Speed increase	83	154	4015	45	43	3.58
Radioactive contamination	27	159	4613	42	39	4.08
Light emission (abnormal)	879	121	2652	36	35	3.46
Light flashes	691	105	2438	34	33	3.18
Unstable steps motion	846	167	4347	46	45	3.71
Electrostatic impact	257	142	2942	39	38	3.74
Motor and/or electrical failure	174	125	3521	37	36	3.47
Cumulative average:						3.63125

The use of three data sources (video, audio, sensors) made it possible to improve the accuracy of determining situations by 26% (Table 2).

**Table 2:** Assessment of situation identification accuracy using data from three data sources.

Situation type and number			Video-	Audio-	Sensors	Altogether	Errors	Accuracy increase
A	Secure situation	1724	62.53%	68.3%	56.9%	73.1%	2.51%	8.06%
B	Threatening situation	1293	56.7%	52.12%	82.1%	86.5%	3.19%	26.61%
C	Emergency situation	947	49.81%	75.23%	93.48%	94.71%	0.38%	44.52%
In total, by average:								26.397%

The sensors give the largest increase in the number of detected events and have the lowest probability of error in determining emergencies. Video processing has the greatest effect in detecting threatening situations (first row of Table 2), and audio processing (62.5% versus 68.3%) has the greatest effect in determining safe situations. However, processing data from all three sources provides more accurate identification of all situations.

To assess the accuracy of determining situations, a sample consisting of 3964 situations was considered.

Based on the data on the system operation during the pilot operation (at the test bench, for six months, from March to September 2021), estimates of the speed of decision-making by the operators were obtained (Table 3).

**Table 3:** Assessment of decision-making efficiency.

Event	Earlier	Now	Results
Help to disabled persons	20–30 min	17–26 min	32.8%
Fire department	5–10 min	3–8 min	19.4%
Ambulance	10–20 min	7–16 min	14.31%
Police	2–5 min	1–4 min	8.431%
Anti-terror services (FSS, etc.)	30–40 min	25–37 min	3.49%
Investigative committee	15–20 min	13–19 min	5.27%
Electricians on duty	3–7 min	1–5 min	46.73%
Plumbers (Hydraulics)	7–15 min	5–12 min	32.41%
Mechanics of escalator service (ES)	2–7 min	1–4 min	61.2%
Support group of ES	2–5 min	1–2 min	73.9%
In total, by average:			29.79%

The new system has made it possible to enhance the performance of decision-making by almost 30% (29.79%). Services for disabled persons, electricians, plumbers and escalator staff (mechanics and support staff) are at the top in the decision-making list regarding the speed of response. These results are because the implementation of the new system has reduced the time to detect threatening and emergency situations and transmit data on them to subway services.

The results of the usability assessment are shown in Table 4.

**Table 4:** Assessments of system usability.

Staff, number		Pro	Cont	Lie	Neutral	Results
Assistant to low mobility groups (disabled)	73	34	17	3	49	34%
Escalator operator	89	41	5	1	54	72%
Subway controller	76	32	19	7	49	26%
Guard	91	28	23	9	49	10%
Mechanics specialist	83	23	19	13	58	8%
Hydraulics specialist	79	26	14	11	60	24%
Electrician	62	21	13	24	66	16%
In total, by average						27.1%

To assess the usability of the system, a standard psychosocial loyalty questionnaire was used with 100-point scales, where 50 points can be scored to express negative attitudes and 50 points to express positive attitudes. Columns in Table 4 indicate: Pro—approving answers, Cont.—dissatisfaction, Lie—false and contradictory answers, Neutral—found it difficult to answer and neutral answers. Questionnaires were completed anonymously, without any personal information, except for the type of position held in the organization. The average ratio of positive to negative responses was 27.1%. The most positive responses were received from escalator operators and MMG assistance employees (assistance for disabled), which can be explained by the appearance of an application on their smartphones that displays operational data on events occurring on the escalator and allows viewing images of the escalator.

## Conclusions

The analysis of existing systems allows us to conclude that they are not capable of providing data processing performance and accuracy of the results to ensure a sufficient level of safety on the escalators. The authors propose an approach to increase the accuracy of detecting and recognizing threatening situations of different types by virtue of intelligent data processing from three sources (video, audio and sensors) and to increase the efficiency of making management decisions. In the interests of this, based on machine learning and recurrent neural networks, a new method and algorithms for managing the safety of passengers on escalators are proposed. The architecture of a promising safety system and implementation of its components for cloud and fog computing environments are developed. Data processing performance enhancement is attained due to the combined use of software in the cloud, fog and on user smartphones. In addition, the usage of a smartphone application provides easier access to the system for the end users.

The results of the experiments demonstrated an accuracy increase in recognizing situations by 8.11% and an increase in data processing performance by almost 4 times when using data from one type of source. Regarding data processing from all three types of sources, the accuracy increases by more than 26%. The speed of decision making by the control operator increases by almost 30%. The usability of the developed application for smartphones is confirmed by the results of psychological questioning of users, according to which the number of positive responses about the application increased by more than 27% compared to those on previously used systems. The proposed approach seems to be a promising one for the solution of other problems in various subject domains. Expanding the scope of the approach application requires further study.

## Data Availability

All the necessary data to repeat the experiments is here: https://github.com/alex1543/subbotin.
